# RNA-Seq–based transcriptome analysis of corneal endothelial cells derived from patients with Fuchs endothelial corneal dystrophy

**DOI:** 10.1038/s41598-023-35468-y

**Published:** 2023-05-27

**Authors:** Tatsuya Nakagawa, Yuichi Tokuda, Masakazu Nakano, Yuya Komori, Naoya Hanada, Theofilos Tourtas, Ursula Schlötzer-Schrehardt, Friedrich Kruse, Kei Tashiro, Noriko Koizumi, Naoki Okumura

**Affiliations:** 1 Department of Biomedical Engineering, Faculty of Life and Medical Sciences, Doshisha University, Kyotanabe, 610-0394 Japan; 2 Department of Genomic Medical Sciences, Kyoto Prefectural University of Medicine, Kyoto, Japan; 3 Department of Ophthalmology, University of Erlangen-Nürnberg, Erlangen, Germany

**Keywords:** Clinical genetics, Gene expression, Genetics research

## Abstract

Fuchs endothelial corneal dystrophy (FECD) is the most common inherited corneal disease. Fibrillar focal excrescences called guttae and corneal edema due to corneal endothelial cell death result in progressive vision loss. Multiple genetic variants have been reported, but the pathogenesis of FECD is not fully understood. In this study, we used RNA-Seq to analyze differential gene expression in the corneal endothelium obtained from patients with FECD. Differential expression analysis of transcriptomic profiles revealed that expression of 2366 genes (1092 upregulated and 1274 downregulated genes) was significantly altered in the corneal endothelium of patients with FECD compared to healthy subjects. Gene ontology analysis demonstrated an enrichment of genes involved in extracellular matrix (ECM) organization, response to oxidative stress, and apoptotic signaling. Several pathway analyses consistently indicated the dysregulation of ECM-associated pathways. Our differential gene expression findings support the previously proposed underlying mechanisms, including oxidative stress and apoptosis of endothelial cells, as well as the phenotypic clinical FECD hallmark of ECM deposits. Further investigation focusing on differentially expressed genes related to these pathways might be beneficial for elucidating mechanisms and developing novel therapies.

## Introduction

Fuchs endothelial corneal dystrophy (FECD) causes severe vision loss and accounts for approximately 40% of all corneal transplantations^1^. Clinical hallmarks of FECD are: (1) excessive production of extracellular matrix (ECM) between the corneal endothelium and Descemet’s membrane (the basement membrane of the corneal endothelium), and (2) damage to corneal endothelial cells (CECs)^2,3^. The ECM forms focal excrescences called guttae, resulting in visual disturbance due to reduced contrast sensitivity and increased glare^4–7^. Corneal endothelial decompensation due to damage to the CECs induces corneal edema, resulting in further severe vision loss due to the loss of corneal transparency^2^. FECD has been accepted as the most common hereditary corneal disorder, as it shows an autosomal dominant pattern of inheritance^8^. However, the causative genes remain unclear, suggesting a need for in-depth studies that make the most of current genomics advances.

One indispensable tool for analyzing gene function is RNA sequencing (RNA-Seq)^9^. Analysis of differentially expressed genes (DEGs) is the most frequent application of RNA-Seq, but RNA-Seq is also suitable for analyzing many aspects of RNA biology, including mRNA splicing and the roles of non-coding and enhancer RNAs. In addition, the emergence of RNA-Seq has introduced the use of RNA-based biomolecules as useful diagnostic, prognostic, and therapeutic tools in various diseases^10,11^. This suggests that RNA-Seq analysis of the corneal endothelium of patients with FECD could be valuable in identifying causative genes. However, our recent repository search revealed only three reports with RNA-Seq datasets.

The first report by Weiben and colleagues appeared in 2018 and described RNA-Seq results for 24 corneal endothelial samples obtained from patients with FECD^12^. However, the authors focused their study on a comparison of differential gene expression between FECD subjects with or without a trinucleotide repeat expansion in the intron of the *TCF4* gene, a mutation with a known association with FECD pathogenesis. Consequently, the paper lacked any analysis of DEGs between patients with FECD and healthy subjects. The second study, by Nikitina and colleagues, generated an RNA-Seq dataset based on 12 patients with FECD and 6 control tissues from eye bank donors, but they did not conduct any further analysis, including enrichment analysis^13^. In the third paper, published in 2020, Chu and colleagues were the first to conduct a comparative pathway analysis of DEGs in the corneal endothelium of patients with FECD versus healthy subjects^14^.

Our main goals in the current study were to obtain an additional RNA-Seq dataset from CECs derived from Caucasian FECD subjects and healthy control subjects to identify DEGs and to conduct enrichment analysis to reveal pathways that are potentially related to the pathophysiology of FECD.

## Results

### Sample information

**Figure 1:**
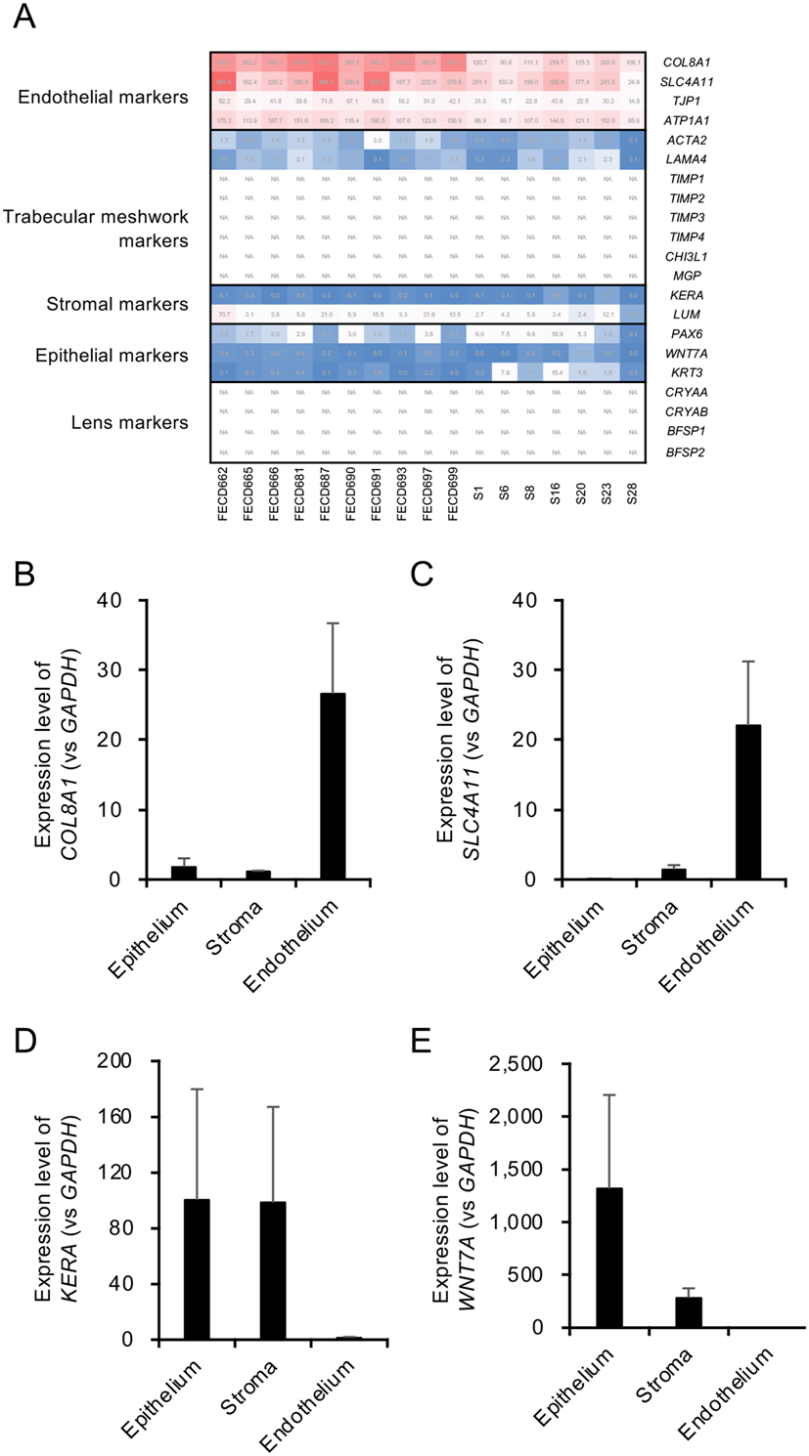
Expression levels of corneal endothelial markers in samples. (**A**) Transcripts per million (TPM) values of representative corneal endothelial markers (*COL8A1*, *SLC4A11*, *TJP1* and *ATP1A1*) were high in corneal endothelial samples. By contrast, the TPM values of trabecular meshwork markers (*ACTA2*, *LAMA4, TIMP1, TIMP2, TIMP3, TIMP4, CHI3L1,* and *MGP*), stromal markers (*KERA* and *LUM*), epithelial makers (*PAX6*, *WNT7A,* and *KRT3*), and lens markers (*CRYAA, CRYAB, BFSP1,* and *BFSP2*) were low or not detectable (indicated as NA). (**B**, **C**) qPCR showed that the expression of corneal endothelial markers *COL8A1* and *SLC4A11* was high in corneal endothelium but limited in the epithelium and stroma. (**D**, **E**) The stromal marker *KERA* and the epithelial marker *WNT7A* were almost undetectable in corneal endothelial samples.

Corneal endothelium of patients with FECD (n = 10) and that of healthy control subjects previously described (n = 7)^15^ were analyzed in this study (Table 1). No significant differences were found for age or sex between the patients with FECD and the control subjects (Table 2). The samples were validated based on the transcripts per million (TPM) values of representative corneal endothelial markers and on the trabecular meshwork, stroma, and epithelium markers (Fig. 1A). The expression levels of corneal endothelial markers (*COL8A1*, *SLC4A11*, *TJP1*, and *ATP1A1*)^16^ were high in corneal endothelial samples, although the expression of trabecular meshwork markers (*ACTA2*, *LAMA4*, *TIMP1*, *TIMP2*, *TIMP3*, *TIMP4*, *CHI3L1*, and *MGP*)^17–21^, stromal markers (*KERA* and *LUM*)^22,23^, epithelium makers (*PAX6*, *WNT7A,* and *KRT3*)^24,25^, and lens markers (*CRYAA, CRYAB, BFSP1,* and *BFSP2*)^26,27^ was low or not detectable. We confirmed the quality of the extracted RNA and the expression of marker genes from additional corneal endothelial samples obtained by stripping Descemet’s membrane from the corneal stroma (this is the same procedure used to collect corneal endothelial samples for RNA-Seq). We found high expression of the endothelial markers *COL8A1* and *SLC4A11* (Fig. 1B,C), but almost no expression of the stromal marker *KERA* or the epithelial marker *WNT7A,* in the endothelial samples (Fig. 1D,E), supporting the purity of the corneal endothelial samples used for the current RNA-Seq study.

**Table 1:** Sample information.

Category	Sample ID	Age	Sex	RIN^†^	Concentration (ng/μl)^‡^	Yield (ng)
Control	S1^#^	69	Female	7.6	13.9	695
Control	S6^#^	62	Female	8.3	21.3	1065
Control	S8^#^	69	Male	7.5	11.9	595
Control	S16^#^	57	Female	7.9	11	550
Control	S20^#^	48	Male	7.7	0.4	20
Control	S23^#^	64	Female	7.9	6.6	330
Control	S28^#^	59	Male	8.8	11.9	595
FECD	FECD662	77	Male	7.5	10.4	520
FECD	FECD681	63	Female	6.8	8.7	435
FECD	FECD693	78	Male	6.4	13.7	685
FECD	FECD697	61	Female	6.7	10.3	515
FECD	FECD665	67	Male	7.1	10.5	525
FECD	FECD666	79	Female	7.4	10.5	525
FECD	FECD687	53	Male	7.0	12.8	640
FECD	FECD690	64	Male	8.2	2.0	100
FECD	FECD691	68	Female	7.6	3.3	165
FECD	FECD699	64	Female	6.9	8.1	405

^†^RNA Integrity Number (RIN) was calculated using Agilent 2100 expert software.

^‡^RNA concentrations were measured by NanoDrop 2000.

^#^These subjects were described in our previous study^15^.

**Table 2:** Demographic data of the patients with Fuchs endothelial corneal dystrophy (FECD) and non-FECD subjects.

	Control (n = 7)	FECD (n = 10)	*P* value
Age (years) (min, max)	61.1 ± 6.8 (48, 69)	67.4 ± 7.9 (53, 79)	0.203^†^
Sex			1.00^‡^
Female	4	5	
Male	3	5	

^†^Mann–Whitney U test.

^‡^Fisher’s exact test.

### Identification and confirmation of DEGs

**Figure 2:**
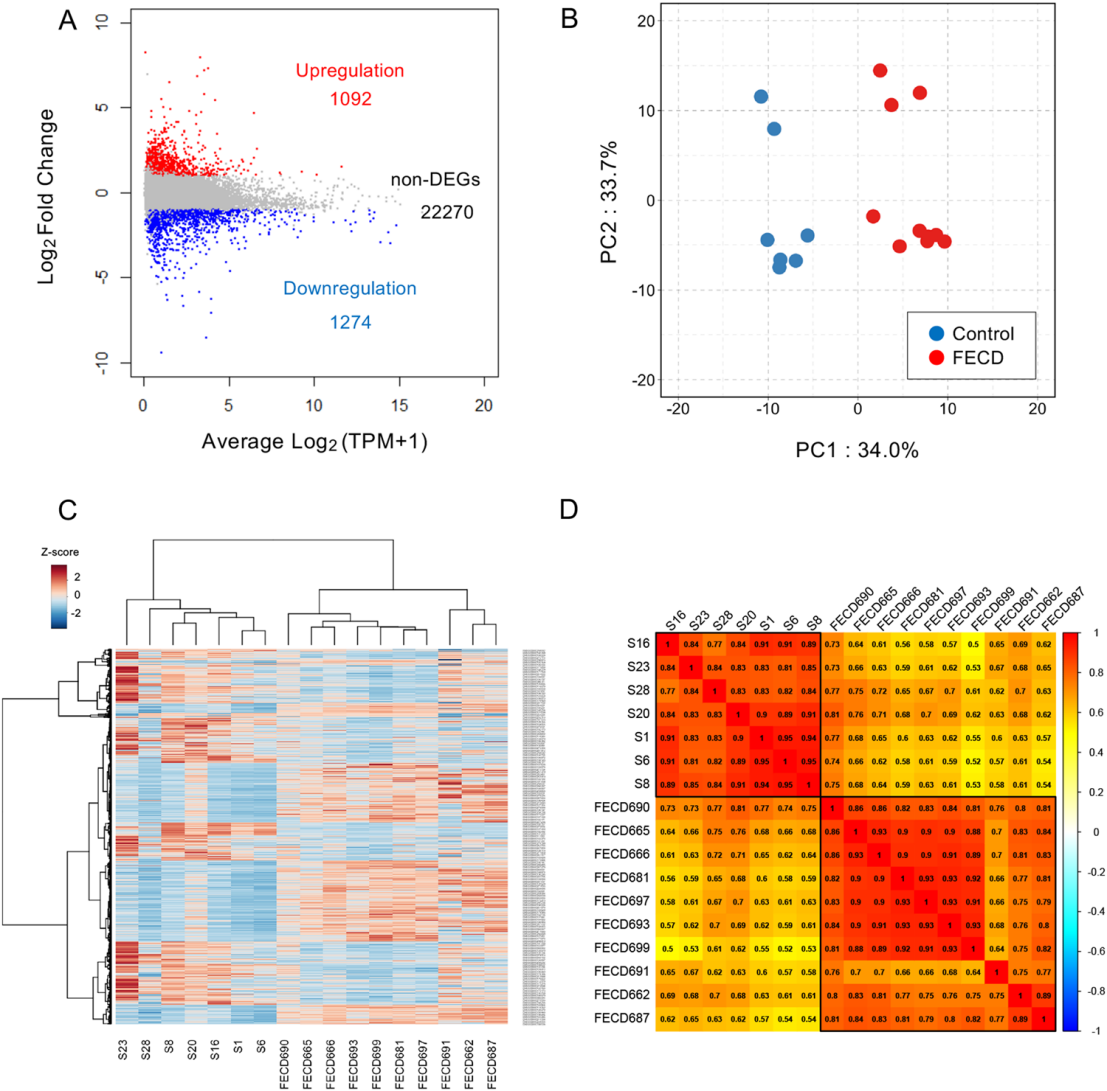
Confirmations of the RNA-Seq data profile. (**A**) MA plot of differentially expressed genes (DEGs) shows 1092 upregulated (in red) and 1274 downregulated (in blue) genes in patients with Fuchs endothelial corneal dystrophy (FECD) from a total of 24,636 expressed genes. The log_2_ fold change between FECD and control samples is plotted on the y-axis and Average Log_2_ (TPM + 1.0) in all samples is plotted on the x-axis. (**B**) Principal component analysis (PCA) reveals the distinct presence of two visual groups in PC1 (x-axis) and PC2 (y-axis). The proportions of variance in PC1 and PC2 are indicated on the x- and y-axes, respectively. Blue and red dots indicate the control and FECD groups, respectively. (**C**) Heatmap shows the relative expression level of genes of the control and FECD groups, confirming that the gene expression pattern of FECD cases is distinct from that of healthy controls. Genes and groups are indicated on the right side and bottom, respectively. Red stripes represent high expression levels, while blue stripes represent low expression levels. (**D**) Correlation matrix utilizing Spearman’s rank correlation coefficients splits the samples into two clusters (control and FECD groups) by Ward's method.

Overall, 24,636 genes were extracted from the 60,164 reference genes through the QC process using the Wald test. This identified 1092 upregulated and 1274 downregulated genes in the FECD samples (2366 DEGs in total) (Supplemental Fig. 1 and Supplementary file 1). The MA plot revealed a global overview and DEG distribution of the gene expression patterns of FECD samples compared to the control samples (Fig. 2A). We then confirmed the influence of DEGs on the FECD and control samples by subjecting the expression data of 2366 DEGs to several analyses.

Principal component analysis (PCA) revealed the presence of two groups, the FECD and control samples (Fig. 2B). A heatmap confirmed a hierarchical clustering of the FECD and control groups based on gene expressions (Fig. 2C). The correlation coefficients also showed high correlations within each group of FECD and control samples. However, the correlation between the FECD and control samples was smaller than the correlations within each group (Fig. 2D). These data profiles demonstrated differences in the gene expression patterns characterized by the DEGs between the FECD and control groups.

### Gene Ontology (GO) enrichment analysis for protein-coding DEGs

**Figure 3:**
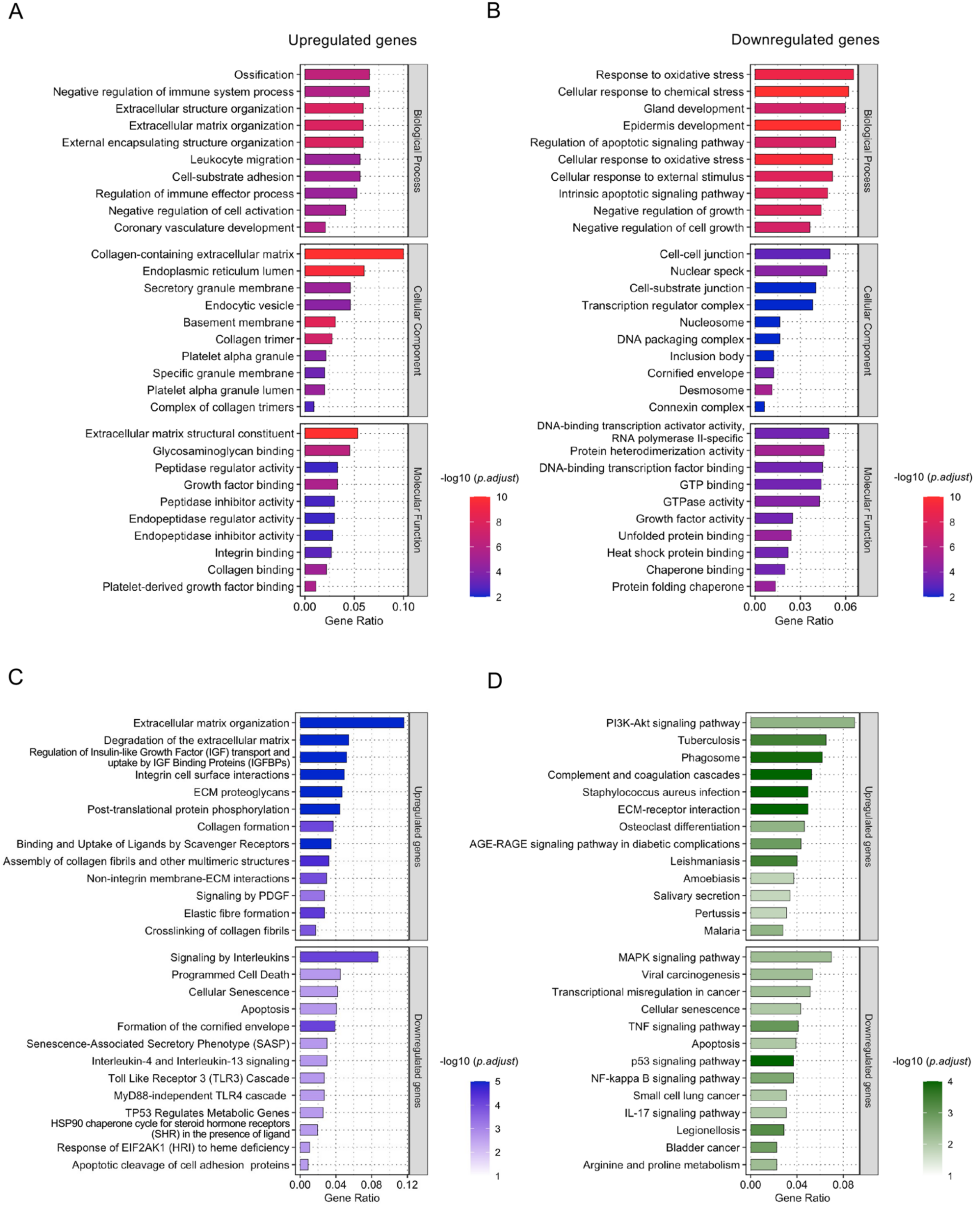
Enrichment analyses of the corneal endothelium of patients with Fuchs endothelial corneal dystrophy (FECD). (**A**) The top GO terms identified by enrichment analysis for upregulated genes. (**B**) The top GO terms identified by enrichment analysis for downregulated genes. The y-axis represents the top 10 GO terms, based on the statistical significance of the alterations in the expression levels of genes in each GO category. Numbers beside the x-axis represent the ratios of altered genes in each GO term. (**C**) The top 13 most enriched Reactome pathways of the differentially expressed genes (DEGs). (**D**) The top 13 most enriched KEGG pathways of the DEGs. The y-axis represents the rank of pathways based on the statistical significance of expression levels for genes in separate to upregulated (upper) and downregulated (lower) genes. The numbers beside the x-axis represent the ratios of altered genes in each pathway. The levels of significance in the enrichment analysis are indicated by their − log_10_ adjusted *P* value and are shown by the bars with the gradient colors.

For GO enrichment analysis, 1706 protein-coding genes, consisting of 696 upregulated and 1010 downregulated genes, were identified in 2366 DEGs by “BioMart.” The top 50 upregulated and downregulated protein-coding genes, based on the log_2_ fold changes between FECD and control samples, are shown in Tables 3 and 4, respectively. The GO analysis revealed that the upregulated genes were associated with the biological processes of extracellular structure organization and ECM organization (Fig. 3A), while the downregulated genes were associated with responses to oxidative stress, epidermis development, and regulation of the apoptotic signaling pathway (Fig. 3B). Upregulated genes were associated with the cellular components of the collagen-containing extracellular matrix, endoplasmic reticulum lumen, and secretory granule membrane (Fig. 3A), while downregulated genes were associated with nuclear specks, cell–cell junctions, and cell–substrate junctions (Fig. 3B). The upregulated genes were associated with the molecular functions of extracellular matrix structural constituents, glycosaminoglycan binding, and peptidase regulator activity (Fig. 3A), while the downregulated genes were associated with nucleoside binding, ribonucleoside binding, and purine ribonucleoside binding (Fig. 3B). The list of GO terms and associated genes is shown in Supplementary file 2.

**Table 3:** Top 50 upregulated protein-coding genes in the corneal endothelium of patients with Fuchs endothelial corneal dystrophy (FECD) compared to non-FECD controls.

Ensembl Gene ID	Gene Symbol	Log_2_ FC (FE/CN)^†^	*P* value^‡^
ENSG00000170373	*CST1*	7.95	2.10 × 10^−10^
ENSG00000244734	*HBB*	7.28	3.57 × 10^−12^
ENSG00000206172	*HBA1*	7.19	1.39 × 10^−10^
ENSG00000188536	*HBA2*	6.86	4.52 × 10^−8^
ENSG00000101441	*CST4*	6.50	2.31 × 10^−8^
ENSG00000135480	*KRT7*	5.79	1.54 × 10^−18^
ENSG00000170369	*CST2*	5.48	2.92 × 10^−4^
ENSG00000115414	*FN1*	5.46	3.73 × 10^−31^
ENSG00000133055	*MYBPH*	5.03	1.42 × 10^−7^
ENSG00000244752	*CRYBB2*	4.89	7.41 × 10^−6^
ENSG00000162706	*CADM3*	4.81	6.32 × 10^−6^
ENSG00000276076	*CRYAA2*	4.77	1.58 × 10^−4^
ENSG00000118785	*SPP1*	4.76	9.50 × 10^−13^
ENSG00000133048	*CHI3L1*	4.65	3.78 × 10^−23^
ENSG00000198734	*F5*	4.56	4.55 × 10^−29^
ENSG00000095970	*TREM2*	4.42	2.83 × 10^−5^
ENSG00000158869	*FCER1G*	4.41	2.29 × 10^−7^
ENSG00000164761	*TNFRSF11B*	4.14	5.39 × 10^−6^
ENSG00000138650	*PCDH10*	4.13	1.47 × 10^−4^
ENSG00000159189	*C1QC*	4.09	4.27 × 10^−8^
ENSG00000019186	*CYP24A1*	4.02	8.25 × 10^−4^
ENSG00000173369	*C1QB*	3.97	3.69 × 10^−7^
ENSG00000177575	*CD163*	3.83	4.44 × 10^−5^
ENSG00000090382	*LYZ*	3.81	5.12 × 10^−4^
ENSG00000132031	*MATN3*	3.80	1.49 × 10^−10^
ENSG00000187800	*PEAR1*	3.70	1.69 × 10^−13^
ENSG00000159212	*CLIC6*	3.67	6.04 × 10^−13^
ENSG00000142173	*COL6A2*	3.60	6.60 × 10^−7^
ENSG00000148677	*ANKRD1*	3.59	1.96 × 10^−3^
ENSG00000213088	*ACKR1*	3.58	1.11 × 10^−6^
ENSG00000129538	*RNASE1*	3.57	6.53 × 10^−4^
ENSG00000183036	*PCP4*	3.55	3.43 × 10^−8^
ENSG00000204472	*AIF1*	3.54	1.72 × 10^−5^
ENSG00000147257	*GPC3*	3.53	4.46 × 10^−7^
ENSG00000205426	*KRT81*	3.51	1.35 × 10^−3^
ENSG00000196136	*SERPINA3*	3.46	3.95 × 10^−10^
ENSG00000078081	*LAMP3*	3.41	3.06 × 10^−12^
ENSG00000165646	*SLC18A2*	3.39	2.20 × 10^−3^
ENSG00000182492	*BGN*	3.39	1.90 × 10^−11^
ENSG00000204287	*HLA-DRA*	3.38	9.80 × 10^−4^
ENSG00000011600	*TYROBP*	3.23	2.12 × 10^−5^
ENSG00000019169	*MARCO*	3.18	4.95 × 10^−3^
ENSG00000176697	*BDNF*	3.16	6.82 × 10^−7^
ENSG00000155659	*VSIG4*	3.15	1.39 × 10^−4^
ENSG00000130208	*APOC1*	3.13	1.06 × 10^−4^
ENSG00000203747	*FCGR3A*	3.05	1.30 × 10^−4^
ENSG00000165168	*CYBB*	3.04	1.28 × 10^−4^
ENSG00000124126	*PREX1*	2.98	1.61 × 10^−6^
ENSG00000166510	*CCDC68*	2.98	6.35 × 10^−10^
ENSG00000164932	*CTHRC1*	2.96	1.81 × 10^−5^

^†^This means Log_2_ Fold Change (FECD/Control).

^‡^*P* value with adjustment was calculated by Wald test DESeq2.

**Table 4:** Top 50 downregulated protein-coding genes in the corneal endothelium of patients with Fuchs endothelial corneal dystrophy (FECD) compared to non-FECD controls.

Ensembl Gene ID	Gene Symbol	Log_2_ FC (FE/CN)^†^	*P* value^‡^
ENSG00000203812	*H2AC18*	− 9.40	1.61 × 10^−2^
ENSG00000187242	*KRT12*	− 8.52	2.34 × 10^−23^
ENSG00000186081	*KRT5*	− 7.10	4.18 × 10^−17^
ENSG00000175793	*SFN*	− 6.67	1.30 × 10^−38^
ENSG00000166426	*CRABP1*	− 6.31	5.02 × 10^−29^
ENSG00000169429	*CXCL8*	− 6.28	1.15 × 10^−13^
ENSG00000186847	*KRT14*	− 6.11	1.45 × 10^−21^
ENSG00000095713	*CRTAC1*	− 6.03	6.68 × 10^−9^
ENSG00000198074	*AKR1B10*	− 5.85	4.50 × 10^−9^
ENSG00000134757	*DSG3*	− 5.40	1.45 × 10^−21^
ENSG00000165474	*GJB2*	− 5.27	4.84 × 10^−9^
ENSG00000206075	*SERPINB5*	− 5.07	9.11 × 10^−9^
ENSG00000163739	*CXCL1*	− 5.04	2.19 × 10^−26^
ENSG00000171346	*KRT15*	− 5.03	6.17 × 10^−10^
ENSG00000186442	*KRT3*	− 5.02	3.96 × 10^−8^
ENSG00000134762	*DSC3*	− 4.96	8.99 × 10^−13^
ENSG00000137440	*FGFBP1*	− 4.89	1.54 × 10^−10^
ENSG00000184292	*TACSTD2*	− 4.73	1.50 × 10^−16^
ENSG00000100292	*HMOX1*	− 4.64	9.38 × 10^−7^
ENSG00000189143	*CLDN4*	− 4.54	1.17 × 10^−16^
ENSG00000165272	*AQP3*	− 4.49	5.81 × 10^−13^
ENSG00000081041	*CXCL2*	− 4.47	2.71 × 10^−11^
ENSG00000136943	*CTSV*	− 4.44	5.84 × 10^−6^
ENSG00000124429	*POF1B*	− 4.41	9.11 × 10^−9^
ENSG00000136244	*IL6*	− 4.39	9.81 × 10^−9^
ENSG00000134760	*DSG1*	− 4.29	1.42 × 10^−10^
ENSG00000171401	*KRT13*	− 4.25	2.48 × 10^−4^
ENSG00000163435	*ELF3*	− 4.18	1.27 × 10^−15^
ENSG00000197632	*SERPINB2*	− 4.18	1.41 × 10^−10^
ENSG00000277586	*NEFL*	− 4.16	4.57 × 10^−5^
ENSG00000143217	*NECTIN4*	− 4.11	4.84 × 10^−9^
ENSG00000135373	*EHF*	− 4.08	6.17 × 10^−10^
ENSG00000109321	*AREG*	− 4.06	7.42 × 10^−4^
ENSG00000177459	*ERICH5*	− 4.05	1.01 × 10^−7^
ENSG00000276903	*H2AC16*	− 3.92	8.00 × 10^−9^
ENSG00000255398	*HCAR3*	− 3.90	1.64 × 10^−8^
ENSG00000141682	*PMAIP1*	− 3.77	6.53 × 10^−4^
ENSG00000137699	*TRIM29*	− 3.77	2.07 × 10^−9^
ENSG00000198535	*C2CD4A*	− 3.77	2.04 × 10^−7^
ENSG00000182782	*HCAR2*	− 3.77	3.28 × 10^−10^
ENSG00000112297	*CRYBG1*	− 3.74	5.45 × 10^−9^
ENSG00000134755	*DSC2*	− 3.67	3.01 × 10^−9^
ENSG00000189334	*S100A14*	− 3.66	1.71 × 10^−7^
ENSG00000108602	*ALDH3A1*	− 3.65	5.70 × 10^−5^
ENSG00000121742	*GJB6*	− 3.63	6.81 × 10^−6^
ENSG00000123975	*CKS2*	− 3.58	3.16 × 10^−19^
ENSG00000196878	*LAMB3*	− 3.54	9.88 × 10^−8^
ENSG00000175592	*FOSL1*	− 3.53	2.16 × 10^−8^
ENSG00000114638	*UPK1B*	− 3.52	6.19 × 10^−6^
ENSG00000180440	*SERTM1*	− 3.47	6.04 × 10^−15^

^†^This means Log_2_ Fold Change (FECD/Control).

^‡^*P* value with adjustment was calculated by Wald test DESeq2.

### Pathway-based enrichment analysis

The Reactome pathway analysis indicated that the upregulated genes were associated with extracellular matrix organization, signaling by receptor tyrosine kinase, and degradation of the extracellular matrix, while the downregulated genes were associated with cellular responses to external stimuli, cellular responses to stress, signaling by interleukins, programmed cell death, and cellular senescence (Fig. 3C). The list of Reactome pathways and associated genes is shown in Supplementary file 3. Conversely, Kyoto Encyclopedia of Genes and Genomes (KEGG) pathway analysis^28,29^ demonstrated that the upregulated genes were associated with the phosphatidylinositol 3-kinase (PI3K)/Akt signaling pathway, tuberculosis, phagosomes, focal adhesions, and ECM-receptor interactions, while the downregulated genes were associated with mitogen-activated protein kinase (MAPK) signaling pathways, apoptosis, the p53 signaling pathway, and the NF-kappa B signaling pathway (Fig. 3D). The list of KEGG pathways and associated genes is shown in Supplementary file 4.

## Discussion

In the current study, a total of 24,636 genes were detected in CECs by RNA-Seq, and 2366 genes were identified as DEGs in FECD (1092 upregulated and 1274 downregulated genes). PCA revealed the presence of two visual groups: control and FECD. GO analysis indicated enrichment of the extracellular structure organization, ECM organization, responses to oxidative stress, and the apoptotic signaling pathway. Consistent with this, the Reactome pathway analysis revealed a dysregulation of ECM-related pathways.

Late-onset FECD, the common form of FECD, typically appears in patients older than 50 years of age, whereas early-onset FECD is a very rare disease and shows a clinically different phenotype^2,3^. A mutation in *COL8A2* has been identified as a cause of early-onset FECD^30^, while late-onset FECD shows an autosomal dominant pattern of inheritance, although sporadic cases are often seen in the clinical setting^3,31^. Genetic linkage analysis of large families with FECD has identified multiple potential chromosomal loci associated with FECD^32–36^, and four genetic mutations, *TCF8*^36^, *SLC4A11*^37,38^, *LOXHD1*^39^, and *AGBL1*^40^, have been proposed as FECD causes. However, these genetic mutations have been found only rarely in other cohorts^31^. For instance, we reported that the single nucleotide polymorphisms (SNPs) in *TCF8*, *LOXHD1*, and *AGBL1* showed no heterogeneity in 36 FECD cases, while three nonsense mutations were detected in *SLCA411*^41^. Therefore, the identification of other causative genetic factors is anticipated for the majority of late-onset FECD cases^42^.

In 2010, Baratz and colleagues reported that several non-coding SNPs, including rs613872 around the transcription factor 4 (*TCF4*) gene on chromosome 18, show a strong association with FECD^43^. The same research group subsequently reported that 79% of the patients with FECD harbored an expansion of CTG trinucleotide repeat ≥ 50, whereas only 3% of non-FECD control subjects harbored this CTG expansion^44^. The high prevalence of the CTG expansion in FECD has been confirmed in multiple ethnic cohorts, with the prevalence depending on ethnicity^41,45–51^. Following those discoveries, the following disease mechanisms induced by CTG repeat expansion have been proposed: (1) dysregulation of *TCF4* transcripts^12,50,52,53^; (2) RNA-mediated toxicity^54–57^; (3) repeat-associated non-AUG dependent (RAN) translation^42,58^; and (4) somatic instability of CTG repeat expansion^59^. Consistent with our previous report^12,50,52,53^, our current RNA-Seq data shows that the expression level of *TCF4* was significantly upregulated in FECD compared to control samples, supporting the existence of dysregulation of *TCF4* transcripts (Supplemental Fig. 2). Although the hypothetical mechanisms have been actively investigated, inspired by the high prevalence of the CTG repeat expansion, the mechanism of FECD in cases that do not harbor the repeat expansion remains unclear. One unanswered question is whether FECD without the repeat expansion has an independent causative genetic basis that does not involve *TCF4* or whether FECD with and without the repeat expansion shares the same basis. This question motivated our present RNA-Seq analysis of the multiple aspects of RNA biology to understand the molecular dysregulation inducing FECD.

In this study, we identified 1706 protein-coding DEGs, including 696 upregulated and 1010 downregulated genes, from a total of 2366 DEGs. Our enrichment analysis demonstrated the involvement of ECM organization, ECM-receptor interactions, and the endoplasmic reticulum lumen in the corneal endothelial transcriptome, as well as oxidative stress, in FECD. The reduced vision associated with FECD arises from the formation of fibrous excrescences (clinically called guttae) and thickening of Descemet’s membrane^4–7^. Indeed, guttae have recently been removed by Descemet’s membrane stripping for the improvement of vision^60–66^.

The observed enrichment of pathways related to ECM in this current study is consistent with the clinical finding that excessive production of ECM plays an important role in vision. The endoplasmic reticulum of the CECs in FECD cases is morphologically changed and further associated with an upregulation of markers of the unfolded protein response (UPR). Engler and colleagues proposed that the UPR plays an important role in the mechanism of FECD^67^. Consistent with this, we showed an accumulation of unfolded proteins in the corneal endothelium of 21 independent subjects with FECD^68^. Our subsequent study, using a cell model established from FECD cases, showed that TGF-β signaling induced a chronic overloading of ECM proteins into the endoplasmic reticulum, with a resulting triggering of the intrinsic apoptotic pathway through the UPR^69^.

In addition, the current findings showed a relationship between FECD and both oxidative stress and the p53 signaling pathway. Many reports suggest an involvement of oxidative stress as a canonical cause of disease pathology^70–74^. For instance, the corneal endothelium in eyes with FECD is susceptible to oxidative DNA damage, which in turn leads to p53-mediated apoptosis that may play a role in the cell death process^71^. Taken together, our current enrichment analysis findings support several of the potential mechanisms proposed to underlie FECD. In the future, researchers can utilize RNA-Seq to generate data regarding gene expression related to identified pathways for further elucidation of the molecular mechanism of FECD.

In the early stage of FECD, the corneal endothelium maintains a polygonal cell morphology, but it shows a drop in cell density and the formation of sporadic guttae in the corneal center^2,3^. By contrast, in the severe stage, the CECs lose their polygonal shape and are transformed into fibroblastic cells^75^. Therefore, we speculate that the DEGs observed here might be induced by two processes: (1) the primary alteration of genes due to FECD and (2) a secondary alteration induced by the wound-healing process due to severe cell death. In the current study, we obtained samples from patients with relatively early-stage FECD; thus, their CECs presumably still had a polygonal morphology. A future study comparing the DEGs between early-stage and severe-stage subjects could be informative to illustrate the primary or secondary alterations in gene expression.

A key limitation of our study is the lack of analysis of CTG trinucleotide repeat expansion in *TCF4*, as this repeat expansion has been viewed as the most likely potential cause of FECD, accounting for 20–80% of occurrences^41,45–51^. Only one report has investigated DEGs in patients with and without the repeat expansion^12^. RNA-Seq using each of three batches of samples in that study showed upregulation of 28 genes and downregulation of 11 genes in patients with the repeat expansion compared to patients without the repeat expansion, but no significantly enriched GO terms were found. Repeating this analysis in a larger number of samples in different cohorts would be worthwhile, as it could provide insights into whether FECD with and without the repeat expansion shares a common genetic cause.

In conclusion, we have generated an RNA-Seq dataset from patients with FECD. Enrichment analysis identified multiple ECM-related pathways that are consistent with the FECD clinical hallmarks of the formation of guttae and the thickened fibrous Descemet’s membrane. The findings also support our previous hypothetical proposal that excessive production of ECM plays a central role in the pathophysiology of FECD through cell death induced by ECM changes and promotion of the UPR. Modulation of ECM dysregulation might be a potential therapeutic modality to counteract guttae formation and CEC death.

## Methods

### Ethics statement

The human tissue used in this study was handled under the guidelines based on the ethical principles of the Declaration of Helsinki. This study was performed according to a protocol approved by the ethical review committee of the Friedrich-Alexander Universität Erlangen-Nürnberg (FAU) (Applied number: 140_20 B), the Doshisha University Ethics Committee for Scientific Research Involving Human Subjects (Applied number: 20009), and the Institutional Review Board of Kyoto Prefectural University of Medicine (Applied number: ERB-G-73). Informed consent to obtain Descemet’s membranes with CECs was acquired from patients with FECD who were scheduled to undergo Descemet's membrane endothelial keratoplasty (DMEK) at FAU. The non-FECD human donor corneas were obtained from CorneaGen (Seattle, WA).

### CECs obtained from the patients with FECD

Descemet’s membranes with CECs were recovered from 10 patients with late-onset FECD (5 males and 5 females of Caucasian descent; age range: 53–79 years) during DMEK, and were stored at 4 °C in a storage medium (Optisol-GS; Bausch & Lomb, Rochester, US-NY) for less than 24 h (Supplemental Fig. 3). Descemet’s membranes with CECs were lysed in 700 μL of QIAzol lysis reagent (Qiagen, Valencia, CA) and homogenized with a vortex mixer for 30 s. Samples were shipped from the FAU to Doshisha University packed in dry ice and then stored at − 80 °C until used for experiments.

### Total RNA preparation

The total RNA of CECs from 10 patients with FECD was isolated by the RNeasy Mini Kit (Qiagen) according to the manufacturer’s protocol, as described in our previous report^15^. Briefly, CECs lysed with QIAzol lysis reagent were thawed at 37 °C, mixed with140 μL chloroform, and centrifuged at 12,000 g at 4 °C for 15 min. The supernatant was collected and mixed with an equal volume of 70% ethanol, followed by concentration using spin columns. The quantity and quality of total RNA were determined using an Agilent 2100 Bioanalyzer with an RNA 6000 Pico Kit (Agilent Technologies, Santa Clara, CA). The quality of total RNA was assessed by determining the RNA integrity number (RIN) using the Agilent 2100 Expert Software (Agilent Technologies).

### RNA-Seq library preparation and data processing

The details of the RNA-Seq experiments by next-generation sequencing (NGS) and the procedure for data processing are described in our previous study^15^. Briefly, the RNA-Seq libraries for NGS were generated with a SMARTer Stranded Total RNA-Seq Kit v2—Pico Input Mammalian (Takara Bio Inc., Shiga, Japan), according to the manufacturer’s instructions and sequenced on a HiScanSQ System (Illumina Inc., San Diego, CA) using a TruSeq SBS Kit v3 (Illumina). The resulting fastq files were aligned to the human reference genome (GRCh38) by STAR version 2.7.3, after quality control (QC) filtering. For the mapped reads, the gene expression analysis was performed using RSEM version 1.3.3, and the resulting read count data and the values of TPM were applied to subsequent analyses. The sequencing, data processing, and basic analyses of NGS data were carried out at the NGS Core Facility of Kyoto Prefectural University of Medicine.

### Identification criteria of DEGs

The control RNA-Seq data from the CECs were derived from non-FECD control subjects, as previously reported^15^. Control samples were obtained from donor corneas derived from 7 donors (3 males and 4 females of Caucasian descent; age range: 48–69 years). The DEGs were identified by comparing the gene expression levels in the FECD samples to those in the control samples using the Wald test in “DESeq2” (Bioconductor version 3.14, https://www.bioconductor.org/) with RSEM, giving data for 60,164 reference genes. In the QC process, genes were excluded if they showed “NA” values in padj by the Wald test, indicating a low expression level, and/or if calculation of the Benjamini–Hochberg adjusted *P* value failed. For the remaining genes, DEGs were defined as the genes with | Log_2_ Fold Change |≥ 1 and adjusted *P* values < 0.05. The gene dispersion was visualized with an MA plot using the default packages of R version 4.1.3 (https://www.r-project.org/).

### Confirmation of the data profile

The data profile was confirmed using PCA, heatmap analysis, and correlation matrix analysis to visualize all DEGs from RNA-Seq results based on TPM values using R version 4.1.3. The PCA and heatmap analyses were performed using the “prcomp” function and “heatmap.2” function, respectively, from the “gplots” library. The “ward.D2” option was also utilized in the “hclust” function for cluster methodology in the heatmap analysis. A correlation matrix was computed using the “cor” function for the calculation of Spearman's rank correlation, and then the “corrplot” function was utilized for a correlogram plot. Note that the PCA and correlation matrix were calculated by adding 1 to the TPM values (TPM + 1) prior to common log transformation. This was done to avoid the failure of the logarithm process due to the TPM value including zero.

### GO enrichment analysis

“BioMart” (version 0.9, http://biomart.org/) was applied for gene ID conversion, and non-coding DEGs were excluded, leaving only protein-coding DEGs for further analyses. The “ClusterProfiler” (version 4.2.2) program with the annotation data package “org.Hs.eg.db” (version 3.8.2) was utilized to generate enrichment results. Significantly enriched GO terms were identified with the threshold of adjusted *P* value < 0.05, and the top GO terms were selected and visualized as graphs using the “ggplot2” package (version 3.3.6) in R. The GO terms were composed of three categories: biological process, cellular component, and molecular function.

### Pathway-based enrichment analysis

Reactome and KEGG pathway analyses^28,29^ were used for pathway-based enrichment analysis. The “ClusterProfiler” program was used for KEGG pathway analysis and the results were illustrated using the “ggplot2” package in R. “ReactomePA” (version 1.38.0) and “ggplot2” were also utilized to conduct Reactome pathway analysis and visualization of the results. Significantly enriched pathways were screened out with a threshold of an adjusted *P* value < 0.05, and the top-ranked pathways with gene ratios were shown as a graph.

### Quantitative real-time PCR

The corneal epithelium, stroma, and endothelium were separately obtained from three corneas of independent non-FECD donors. First, an approximately 10 mm diameter of Descemet’s membrane, including the corneal endothelium, was mechanically separated from the peripheral area to avoid contamination of the trabecular meshwork, and Descemet’s membrane, including the corneal endothelium, was peeled off from the corneal stroma. The corneal epithelium and stroma were then recovered separately. Total RNAs were extracted from those samples using an RNeasy Mini Kit (Qiagen). Briefly, the samples were lysed with a QIAshredder and applied to spin columns (Qiagen) with ethanol. Total RNA was eluted from columns, and cDNA was synthesized using a master mix (SuperScript VILO Master Mix; Thermo Fisher Scientific Inc., Waltham, MA). A real-time PCR system (QuantStudio 3; Applied Biosystems) was utilized for quantitative real-time PCR (qPCR). The gene expression levels were calculated by the delta-delta Ct method. The following probes (Thermo Fisher Scientific, Inc.) were used in this study: *COL8A1* (Hs00156669_m1), *SLC4A11* (Hs00984689_g1), *KERA* (Hs00559942_m1), and *WNT7A* (Hs01114990_m1). The *GAPDH* (Hs02786624_g1) was used for the normalization of gene expression levels. All samples were analyzed in duplicate.

## Supplementary Information

Supplementary Figure 1.

Supplementary Figure 2.

Supplementary Figure 3.

Supplementary Information 4.

Supplementary Information 5.

Supplementary Information 6.

Supplementary Information 7.

## Data Availability

All raw fastq files produced by RNA-Seq for patients with FECD were deposited in the DNA Data Bank of Japan (DDBJ) Sequence Read Archive (DRA) under the Accession ID: DRA015078 (https://ddbj.nig.ac.jp/resource/sra-submission/DRA015078) and Genomic Expression Archive (GEA) under the Accession ID: E-GEAD-564 (https://ddbj.nig.ac.jp/public/ddbj_database/gea/experiment/E-GEAD-000/E-GEAD-564). The data details for the healthy control subjects were described in our previous study^15^.
