# Supplementary material for: RNA-Seq–based transcriptome analysis of corneal endothelial cells derived from patients with Fuchs endothelial corneal dystrophy
Source: Sci Rep. 2023 May 27;13:8647. doi: 10.1038/s41598-023-35468-y (PMC10224979; doi:10.1038/s41598-023-35468-y)
Supplement: Supplementary file 1 — Supplementary Figure 1. [file 41598_2023_35468_MOESM1_ESM.pdf]

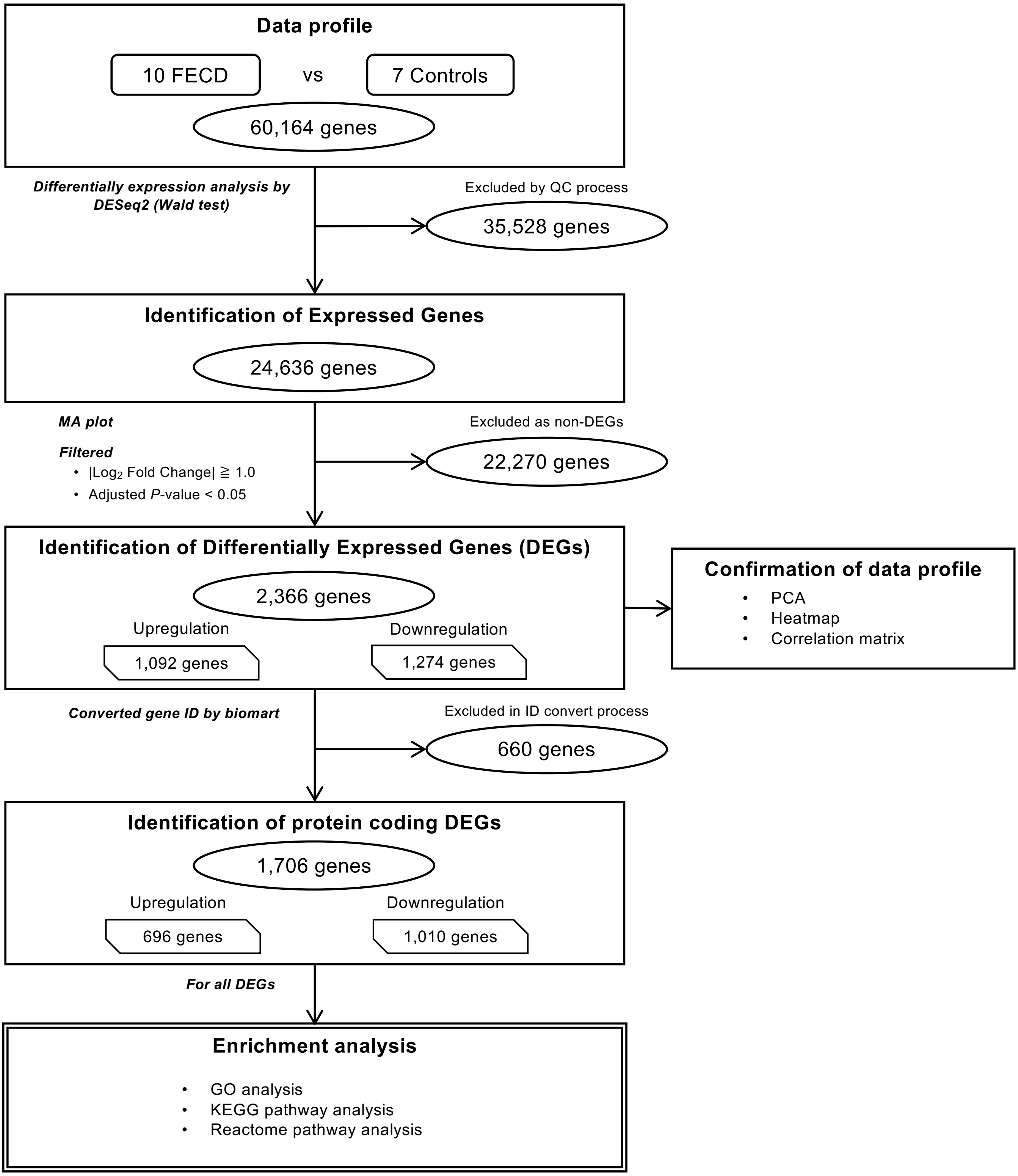

Supplemental figure 1. The workflow of RNA-Seq data analysis.
The 60,164 reference genes in the raw data profile were subjected to differential analysis by DESeq2 software, which identified 24,636 genes as expressed genes. A total of 2,366 differentially expressed genes (DEGs) were selected based on our criteria. After excluding non-coding genes, 1,706 protein-coding-DEGs were used in the enrichment analysis.
