# Supplementary material for: RNA-Seq–based transcriptome analysis of corneal endothelial cells derived from patients with Fuchs endothelial corneal dystrophy
Source: Sci Rep. 2023 May 27;13:8647. doi: 10.1038/s41598-023-35468-y (PMC10224979; doi:10.1038/s41598-023-35468-y)
Supplement: Supplementary file 2 — Supplementary Figure 2. [file 41598_2023_35468_MOESM2_ESM.pdf]

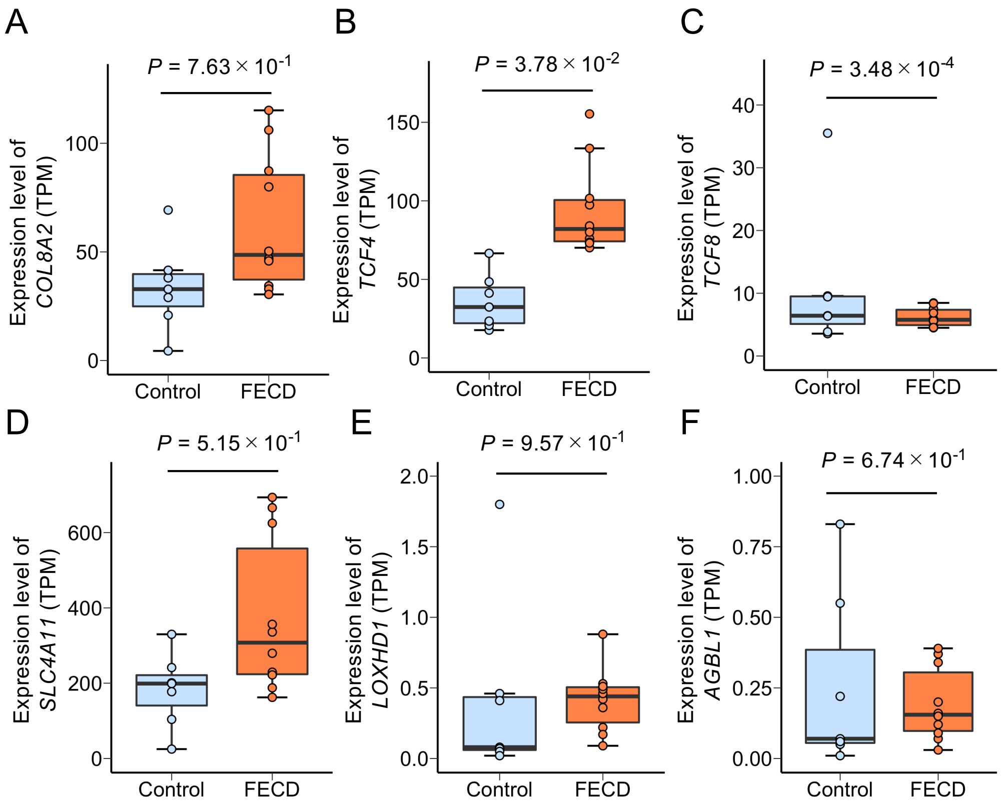

Supplemental figure 2. The expression level of genes potentially related to Fuchs endothelial corneal dystrophy (FECD)

A. *COL8A2* (a point mutation resulting in p.L450W substitution induces early-onset FECD) showed a tendency toward upregulated expression in the corneal endothelium of patients with FECD compared to control subjects, although the difference was not statistically significant.

B. Expression of *TCF4* (intronic trinucleotide repeat expansion—the most frequent mutation associated with late-onset FECD) was significantly upregulated in FECD subjects compared to healthy controls.

C. Expression of *TCF8* (rare mutation potentially related to late-onset FECD) was slightly lower in FECD cases than in controls.

D-F. Expression of *SLC4A11*, *LOXHD1*, and *AGBL1* (a rare mutation potentially related to late-onset FECD) was not significantly different between FECD cases and controls. The *P*-values with Benjamini-Hochberg adjustment were calculated by Wald test DESeq2.
