# Supplementary material for: RNA-Seq–based transcriptome analysis of corneal endothelial cells derived from patients with Fuchs endothelial corneal dystrophy
Source: Sci Rep. 2023 May 27;13:8647. doi: 10.1038/s41598-023-35468-y (PMC10224979; doi:10.1038/s41598-023-35468-y)
Supplement: Supplementary file 3 — Supplementary Figure 3. [file 41598_2023_35468_MOESM3_ESM.pdf]

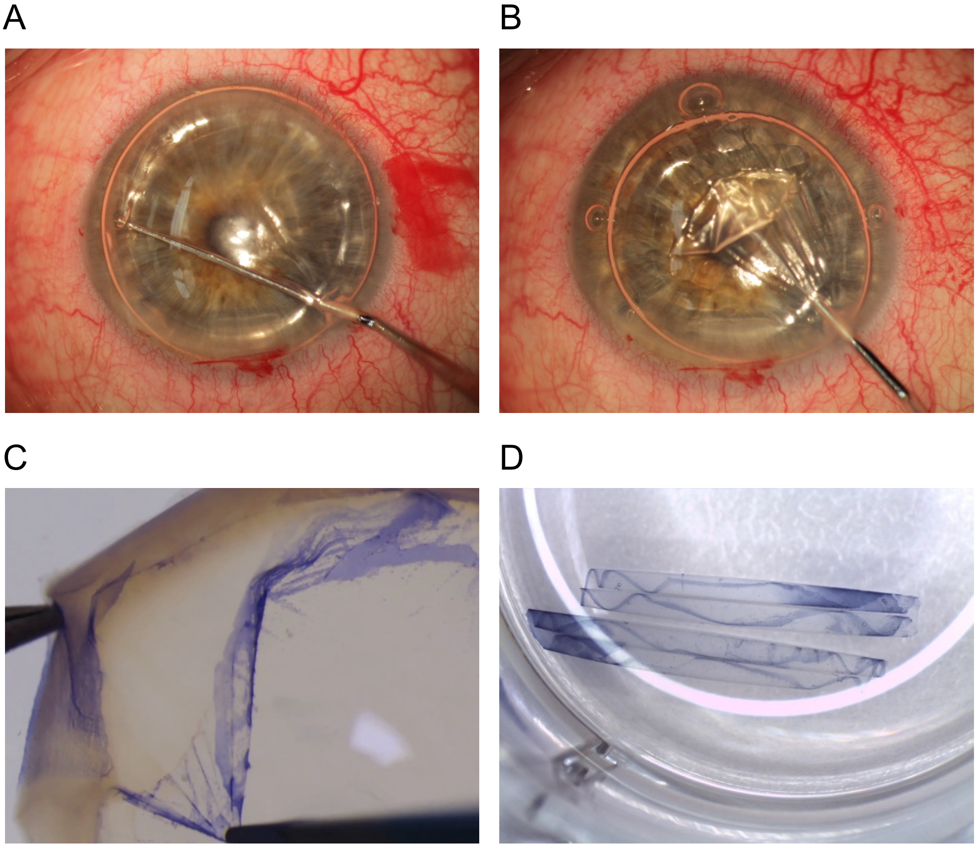

Supplemental figure 3. Sample acquisition of Descemet’s membranes from patients with Fuchs endothelial corneal dystrophy (FECD)

Descemet’s membranes with corneal endothelial cells (CECs) were recovered from patients with late-onset during Descemet's membrane endothelial keratoplasty (DMEK). Descemet’s membrane was isolated as approximately 8 mm in diameter round shape (A) and peeled off from the corneal stroma (B). The removed Descemet’s membrane can be considered to include only the corneal endothelium and no other types of cells.

For obtaining control samples, the stroma, trabecular meshwork, and conjunctiva were removed at the peripheral clear site. Then, Descemet’s membrane, including only the corneal endothelial cells, was peeled off from the corneal stroma (C). The Descemet’s membrane is considered to include only CECs (D).
